# Zein-Layered Hydroxide Biohybrids: Strategies of Synthesis and Characterization

**DOI:** 10.3390/ma13040825

**Published:** 2020-02-11

**Authors:** Ana C. S. Alcântara, Margarita Darder, Pilar Aranda, Eduardo Ruiz-Hitzky

**Affiliations:** Materials Science Institute of Madrid (ICMM), CSIC, c/Sor Juana Inés de la Cruz 3, Cantoblanco, 28049 Madrid, Spain; ana.alcantara@ufma.br (A.C.S.A.); eduardo@icmm.csic.es (E.R.-H.)

**Keywords:** zein, layered double hydroxides, layered single hydroxides, intercalation, biohybrids

## Abstract

This work constitutes a basic study about the first exploration on the preparation of biohybrids based on the corn protein zein and layered metal hydroxides, such as layered double hydroxides (LDH) and layered single hydroxides (LSHs). For this purpose, MgAl layered double hydroxide and the Co_2_(OH)_3_ layered single hydroxide were selected as hosts, and various synthetic approaches were explored to achieve the formation of the zein-layered hydroxide biohybrids, profiting from the presence of negatively charged groups in zein in basic medium. Zein-based layered hydroxide biohybrids were characterized by diverse physicochemical techniques such as X-ray diffraction (XRD), Fourier transform infrared spectroscopy (FTIR), thermogravimetric analysis/differential thermal analysis (TG/DTA), solid state ^13^C cross-polarization magical angle spinning nuclear magnetic resonance (CP-MAS NMR), field emission-scanning electron microscopy (FE-SEM), transmission electron microscopy (TEM), etc., which suggest that the different synthesis procedures employed and the anion located in the interlayer region of the inorganic host material seem to have a strong influence on the final features of the biohybrids, resulting in mixed, single intercalated, or highly exfoliated intercalated phases. Thus, the resulting biohybrids based on zein and layered hydroxides could have interest in applications in biomedicine, biosensing, materials for electronic devices, catalysis, and photocatalysis.

## 1. Introduction

The interest in the development of bionanocomposite materials based on the interaction of inorganic nanoparticulated solids and biopolymers is a topic of great relevance in the development of bioplastics as well as in other areas of application [1]. In this way, layered clays and other related solids, such as layered double hydroxides (LDHs), have been combined with biopolymers, typically polysaccharides and in minor extension proteins, to produce functional materials for a large variety of uses: food-packaging, adsorbents, drug delivery systems, scaffolds for tissue engineering applications, sensors and biosensors, etc. [2,3,4]. It is well known that the combination of proteins with solid surfaces is an important process, not only due to their fundamental aspects of interaction, but also due to the variety of potential applications, from natural biochemistry to biotechnological applications [5]. In this sense, many studies have focused on the interaction of this type of biomacromolecules with layered solids in order to obtain biological–inorganic hybrid compounds that can be used in advanced applications from biocatalysts to synthetic engineering and biosensors, where the investigation of the interaction between both entities at the molecular level is essential to achieve the desired properties [6]. However, the majority of publications reporting biohybrids based on peptides and proteins such as gelatin [7,8], soy protein [9,10], or hemoglobin [11] are assembled to smectite clays, with preference on the montmorillonite aluminosilicate, following cation exchange mechanisms. In this way, it is worth mentioning the pioneering research on protein–clay interactions, which explored the adsorption of the structural protein gelatin, derived from collagen, in the interlayer space of smectites [12]. Nevertheless, the use of other layered solids, such as layered metal hydroxides of the type of LDH and layered single hydroxide (LSH), is attracting more and more interest due to their low cost, high purity, versatility, and numerous applications in diverse areas [4,13,14]. 

**Figure 1:**
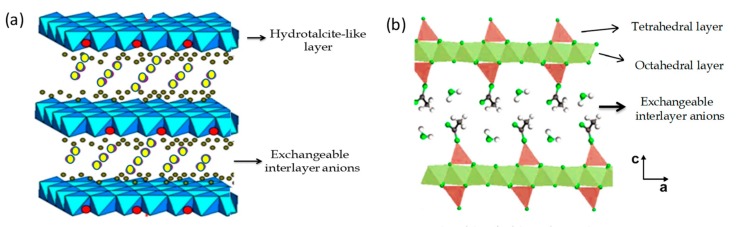
Schematic representation of the crystal structure of (**a**) typical layered double hydroxide (LDH) and (**b**) layered single hydroxide (LSH) layered solids.

LDHs (Figure 1a), also known as hydrotalcite-like compounds or anionic clays, are widely used as catalysts or catalyst precursors, adsorbents, components in modified electrodes, as inorganic host matrices for drug or herbicide release in medical and environmental applications, or as nanofillers of polymers [13,15,16,17,18,19,20,21,22,23,24,25,26]. LDHs can be prepared easily following several synthetic approaches, such as direct anion-exchange and coprecipitation or “co-organized assembly,” commonly used in the preparation of LDH-based biohybrid materials [27]. In this sense, LDHs such as those based on MnAl, NiAl, and ZnCr have been used as hosts of low molecular weight species such as phenylalanine (Phe) amino acid, which was intercalated by the co-precipitation method [28]. The work reported by Nakayama and co-authors [29], describing the intercalation of amino acids and various peptides into a MgAl-LDH by reconstruction from the layered MgAl oxide precursor, is also noteworthy. In that case, the intercalation process was greatly influenced by the type of side chain, structure, and physicochemical properties of the involved amino acid [5,29]. This same procedure was followed to intercalate biomacromolecules like the casein protein into CaAl-LDH, where it was found that β-casein is more easily intercalated than α-casein [30]. Although the intercalation of polysaccharides, oligonucleotides, and other biopolymers in LDHs [4,25,31,32,33,34] is well known, examples of the incorporation of proteins and related biomolecules is still scarce. On the other hand, LSH (Figure 1b) has an identical structure to LDH, but in this case, the inorganic layers are composed of only one type of metal cation, where the anions are coordinated to the in-plane metal ions and the inorganic layers are essentially neutral [21,35]. In spite of this, LSH can also undergo anion-exchange reactions, as occurs in LDH solids, and the LSH-based hybrids are formed by replacing the exchangeable interlayer anions in the LSH lattice with negatively charged organic molecules [22]. It is worth mentioning that, in this case, the metal–anion bond results in a strong interaction between molecules intercalated between the layers and the metal inorganic network [36]. In addition to the ion-exchange reaction, the co-precipitation method is also a procedure usually employed to prepare hybrids based on LSH, resulting in highly promising matrices for the development of new magnetic and optically active hybrids [22,37,38]. Among them, the development of functional biohybrid compounds based on the intercalation of a series of short length peptides in the intracrystalline space of Cu(II) and Co(II) LSH is a remarkable example [36]. 

Within this perspective, this work is focused on the development of biohybrids based on a hydrophobic globular protein, zein, and layered metal hydroxides. Zein is a protein from maize, found as a mixture of protein complexes, and α-zein is the most abundant fraction. This protein presents hydrophobic properties around 50 times higher than those of albumin, γ-globulin, and fibrinogen of bovine blood, which are due to its characteristic amino acid sequence [39]. As a result of the large content of hydrophobic residues in zein, this protein is soluble in pure water only if the pH is raised above 11, likely due to the ionization of the phenolic groups from the tyrosine and because of the presence of glutamic acid residues [40]. Recently, we reported the effective intercalation of zein into Na–montmorillonite, where the cooperative role of the protein components and its solubility were carefully studied in the developed new biointerfaces [41]. Based on these premises, the synthesis of new biohybrid materials based on zein and layered hydroxides can be explored, taking advantage from the presence of negatively charged groups in zein in alkaline medium, when the pH of the solution is raised above its isoelectric point. Thus, this work aims to be the first attempt to study the formation of biohybrids resulting from assembling zein to a 2:1 MgAl-LDH and a Co_2_(OH)_3_ LSH. The main results of the synthesis and physico-chemical characterization of these new zein-layered hydroxides are described. The resulting zein-based biohybrids could show potential interest, for instance, in biomedical applications or surface chemistry processes, such as catalysis or photocatalysis.

## 2. Materials and Methods 

### 2.1. Starting Materials and Reagents

Zein (Z) from corn and Co(CH_3_COO)_2_·4H_2_O (≥ 99%) were purchased from Sigma-Aldrich (Tres Cantos, Spain). Absolute ethanol and MgCl_2_·6H_2_O (≥ 99%) were purchased from Panreac (Castellar del Vallés, Barcelona, Spain). AlCl_3_·6H_2_O (≥ 99%), Mg(NO_3_)_2_·6H_2_O (≥ 99%), NaOH (≥ 98%) were obtained from Fluka (Madrid, Spain) and Al(NO_3_)_3_·9H_2_O (≥ 99%) from Merck (Darmstadt, Germany). Deionized water (resistivity of 18.2 MΩ cm) was obtained with a Maxima Ultrapure Water from Elga (Celle, Germany).

### 2.2. Synthesis of the Layered Hosts

#### 2.2.1. Synthesis of MgAl-Layered Double Hydroxides with Different Inorganic Anions in the Interlayer Space

A mixture of MgCl_2_·6H_2_O (17.5 mmol) and AlCl_3_·6H_2_O (8.74 mmol) was dissolved in 250 mL of decarbonated bi-distilled water to prepare the 2:1 MgAl–Cl LDH. This aqueous solution was dripped with a peristaltic pump to 100 mL deionized water, keeping the mixture under a nitrogen flow to remove CO_2_. Simultaneously, 1 M NaOH solution was also added dropwise to the aqueous mixture through an automatic dispenser (Dosimat 765 with an 806 Exchange Unit, from Metrohm (AG, Herisau, Switzerland) controlled by a 781 pH/Ion Meter (Metrohm) to keep a constant pH of 11.0 ± 0.1 during the synthesis. The resulting dispersion was kept under magnetic stirring and nitrogen flow for 24 h. The solid product was separated by centrifugation, washed three times with bi-distilled and degassed water, and dried at 60 °C overnight. The [Mg_0.67_Al_0.33_(OH)_2_]Cl_0.33_·nH_2_O] LDH was denoted as MgAl–Cl LDH.

The 2:1 MgAl–NO_3_ LDH nitrate was also prepared by an analogous method to that described for the MgAl–Cl LDH, except that Mg(NO_3_)_2_·6H_2_O (17.5 mmol) and Al(NO_3_)_3_·9H_2_O (8.74 mmol) were used as the source of magnesium and aluminum. The [Mg_0.69_Al_0.34_(OH)_2_] NO_3 0.34_·nH_2_O] LDH was denoted as MgAl–Nit LDH.

The 2:1 Mg:Al LDH containing CO_3_^2−^ ions (Mg_2_Al-Carb) was prepared following the method described above for Mg_2_Al–Cl LDH, including the same salts and their respective concentration, except that in the different stages of synthesis the nitrogen flow was not employed and the precipitation pH was 9.0 ± 0.1, adjusted with 0.2 M Na_2_CO_3_ aqueous solution. The [Mg_0.67_Al_0.33_(OH)_2_] CO_3 0.33_·nH_2_O] was denoted as MgAl-Carb LDH. 

#### 2.2.2. Synthesis of Co-Layered Single Hydroxide 

The procedure described by Si et al. [36] was followed to synthesize the Co_2_(OH)_3_(CH_3_COO)·H_2_O LSH. In this procedure, 0.02 mol of Co(CH_3_COO)_2_·4H_2_O was dissolved in 100 mL of water, maintaining the reaction medium at 70 °C under stirring and nitrogen flow. A volume of 0.1 M NaOH (30 mL) mixed with 60 mL of ethanol–water (50%v/v) was added slowly to the previous solution under vigorous stirring. The resulting dispersion was kept under stirring for other 30 min, then centrifuged and washed with water and ethanol to remove the unreacted reagents, and finally dried at 40 °C. The obtained layered material was denoted as Co-LSH. 

### 2.3. Zein-Layered Double Hydroxides 

**Figure 2:**
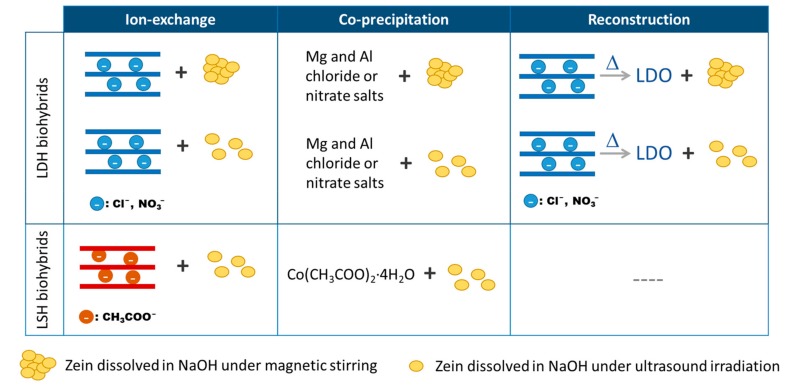
Schematic representation of the routes of synthesis explored in the preparation of the zein-based LDH and LSH biohybrids.

For the preparation of the zein-layered double hydroxide (Z-LDH) biohybrids, three routes of synthesis were employed (Figure 2).

#### 2.3.1. Ion-Exchange Method 

In the ion exchange method used to prepare the zein-LDH biohybrids, 0.5 g of zein was initially dissolved in 0.1 M NaOH (50 mL) through magnetic stirring or by means of an ultrasound tip (VC750 Sonics Vibra-Cell, Newtown, CT, USA, operating at 20KHz). This ultrasound treatment was carried out with a tip of 13 mm diameter and applying intermittent pulses of 10 s followed by a standby step of 10 s, up to a total applied energy of 60 kJ/0.5 g of zein. After zein solubilization, the pH of the solution was adjusted to 11 by addition of 0.1 M HCl and it was slowly added to a dispersion containing 2 g of MgAl–Cl or MgAl–Nit LDH freshly prepared in 50 mL of degassed bi-distilled water. The pH of the system was adjusted back to 11 and then kept under magnetic stirring at 50 °C and N_2_ flow for 4 days. The solid product was recovered by centrifugation, rinsed with distilled water and dried at 40 °C. The resulting hybrid materials derived from MgAl–Cl and MgAl–Nit LDH were denoted as Z-LDH-Cl_ie and Z-LDH-Nit_ie, respectively, when zein dissolved under magnetic stirring was used. Those materials prepared with sonicated zein were denoted as Z-LDH-Cl_ie-US and Z-LDH-Nit_ie-US.

#### 2.3.2. Co-Precipitation Method

Zein was intercalation into the MgAl–Cl or MgAl–NO_3_ LDH by the co-precipitation method by adding slowly 250 mL of an aqueous solution containing the Mg and Al salts to 100 mL of a zein solution (0.5 g of protein/100 mL of 0.1 M NaOH), previously prepared by ultrasonication or magnetic stirring, as previously described for the ion-exchange method. The pH was controlled to 11.0 and the system was kept under magnetic stirring and N_2_ atmosphere for 24 h at room temperature. The solid fraction was separated and dried as previously described for the ion-exchange method. The biohybrid materials prepared by co-precipitation in chloride and nitrate salts and using zein dissolved by magnetic stirring were labeled as Z-LDH-Cl_cppt and Z-LDH-Nit_cppt, respectively, while those obtained from sonicated zein were labeled as Z-LDH-Cl_cppt-US and Z-LDH-Nit_cppt-US, respectively. 

#### 2.3.3. Reconstruction Method

The reconstruction method followed to synthesize the Z-LDH biohybrids was performed by means of calcination-rehydration reaction, where 1 g of LDH MgAl containing carbonate ions in the interlamellar space (MgAl–CO_3_ LDH) was calcined in a muffle furnace at 500 °C for 5 h in order to form the corresponding dehydrated MgAl layered double oxide (MgAl LDO). Zein (0.5 g) was dissolved in 50 mL of 0.1 M NaOH through magnetic stirring or by ultrasonication, as described for the other preparation methods, and the pH of the zein solution was adjusted to 11. The MgAl LDO was added to 50 mL of the zein solution under magnetic stirring and N_2_ flow. The solid product was separated by centrifugation, washed and dried of analogous manner as described for ion-exchange and coprecipitation methods. The hybrid material obtained from this synthesis was labeled as Z-LDH_rec or Z-LDH_rec-US when zein was dissolved by magnetic stirring or by ultrasounds, respectively. 

### 2.4. Zein-Layered Single Hydroxide 

The preparation of zein-layered single hydroxide (Z-LSH) biohybrids was carried out by means of the following two synthetic routes. 

#### 2.4.1. Ion-exchange Method 

The first step in the preparation of zein-LSH by ion-exchange was the solubilization of zein (0.5 g) in 15 mL of 0.1 M NaOH with an ultrasound tip, applying a total energy of 60 kJ/0.5 g of zein. Then, a water–ethanol mixture (50% v/v) was added to the previously prepared zein solution and the pH was adjusted at 8.2. Subsequently, 0.5 mmol of Co-LSH were added to the zein solution under vigorous stirring. The resulting dispersion was stirred at 70 °C under constant N_2_ atmosphere for 24 h. Then, the resulting solid was centrifuged, washed thoroughly with water and ethanol, and dried at 40 °C. The zein-LSH hybrid material obtained by the ion exchange method was denoted as Z-LSH_ie-US.

#### 2.4.2. Co-precipitation Method 

The procedure followed to prepare the zein-LSH biohybrid is similar to that described for the synthesis of the Co-LSH material, except that, in this case, 30 mL of 0.1 M NaOH were used to dissolve 0.25 g of zein. This alkaline zein solution was ultrasonicated using an ultrasound tip, up to a total applied energy of 60 kJ. After adding 60 mL of ethanol–water (50% v/v) to the alkaline zein solution, the resulting mixture was added slowly to the aqueous cobalt solution, and the reaction was kept under magnetic stirring and N_2_ flow for 30 min. The resulting solid was isolated and dried as described for the ion-exchange method. The hybrid material obtained by this procedure was labeled as Z-LSH_cppt-US.

### 2.5. Characterization 

FTIR spectra of samples in film form or diluted in KBr as pellets were recorded from 4000 to 250 cm^−1^ (2 cm^−1^ resolution) with a FTIR spectrophotometer BRUKER IFS (Billerica, MA, USA) 66v/S. CHNS elemental chemical microanalysis of samples was determined in a Perkin-Elmer 2400 analyzer (Waltham, MA, USA). Solid-state ^13^C CP-MAS-NMR spectra of samples spun at 10 KHz were obtained in a Bruker Avance 400 spectrometer (Billerica, MAs, USA), using a contact time of 2 ms and a period between successive accumulations of 5 s. The number of scans was 800 and chemical shift values were referenced to tetramethylsilane. Surface morphology was observed by FE-SEM with a FEI-NOVA NanoSEM 230 (Eindhoven, Netherlands) microscope equipped with a Type SDD Apollo 10 EDAX detector (Leicester, UK), which allowed semi-quantitative analysis of elements. The equipment allows the direct observation of samples adhered on a carbon tap without requirement of any conductive coating on the surface. For the TEM images (Philips Tecnai 20, Amsterdam, Netherlands, operating at 200 kV), the biohybrids were previously embedded in epoxy resin and then cut in very thin sections using an ultramicrotome (LEICA EM UC6, Wetzlar, Germany) equipped with a diamond blade.

### 2.6. SDS-PAGE

The sodium dodecyl sulfate-polyacrylamide gel electrophoresis (SDS-PAGE) technique was performed according to Cabra and co-workers [42], where aliquots of 7.5 µL containing approximately 30 µg of zein solubilized in 0.1 M NaOH under an ultrasound tip (total applied energy of 10, 30 or 60 kJ with 10 s on/off pulses) were re-suspended in equal volumes of deionized water and buffer (0.125 M Tris–Cl, 4% SDS, 20% glycerol, 10% 2-mercaptoethanol (BME), and bromophenol blue 0.01%, pH 6.4). The polyacrylamide gels at 20% were silver-stained for band visualization.

## 3. Results and Discussion

Different routes of synthesis (Figure 2) were evaluated for the preparation of zein-layered hydroxide biohybrid materials in order to achieve the intercalation of this protein in the inorganic host substrate. These synthetic procedures, involving a MgAl LDH containing different interlayer anions and zein protein solubilized in alkaline solution under different experimental conditions (magnetic stirring or ultrasonication), were first tested with the aim to achieve fully zein intercalated LDH solids.

**Figure 3:**
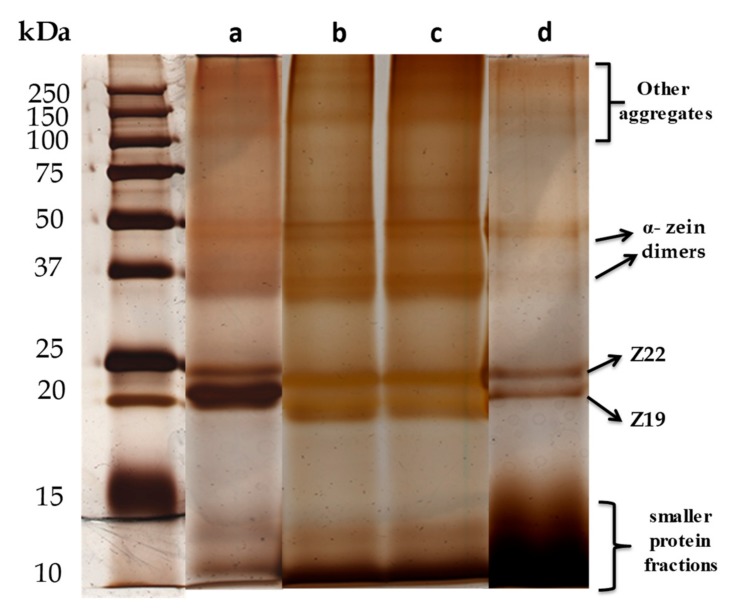
SDS-PAGE profiles in 20% acrylamide of zein after being dissolved in 0.1 M NaOH by (a) magnetic stirring, and under ultrasonication (US) applying total energies of (b) 10, (c) 30, and (d) 60 kJ/0.5 g of zein. The gel was silver stained.

Given that alkaline media treatments ensure the presence of negative charges in the zein structure, NaOH solution of pH 11 was selected for the solubilization of zein, as this medium is also favorable for the synthesis of many LDH [40]. However, it is reported in the literature that alkaline treatment in zein provokes a deamidation of the glutamine amino acid, together with the possible formation of alkali salts of the phenolic-hydroxyl groups in tyrosine [43,44,45]. According to the electrophoresis results (Figure 3), the alkaline treatment of zein breaks the protein in smaller fractions of protein size between 14 and 10 kDa, although the presence of the Z19 and Z22 monomers and α-zein dimers was still evidenced (Figure 3a). Aiming at increasing the number of smaller fractions of zein that can penetrate more easily in the interlayer space of the LDH, zein dissolved in 0.1 M NaOH was submitted to ultrasonication (US) treatment, applying a total energy of 10, 30 and 60 kJ/0.5 g of zein with the help of an ultrasound tip. In this case, the SDS-PAGE analysis of the alkaline-treated zein samples disaggregated under energies of 10 and 30 kJ (Figure 3b,c, respectively), shows a very similar distribution of protein fractions, which are also analogous to that of alkaline-treated zein prepared with no US, just under magnetic stirring (Figure 3a). On the contrary, alkaline-treated zein ultrasonicated at higher energies (i.e, 60 kJ) seems to undergo major changes, showing in the SDS-PAGE the presence of the characteristic bands at 22 kDa and 20 kDa corresponding to the α-helix structure, together with a larger number of smaller protein fractions between 15 and 10 kDa (Figure 3d). In addition to the hydrolysis reactions taking place at basic conditions [46], the presence of small protein fractions may also be attributed to the breakdown of protein aggregates by the high US energy applied in the solubilization process. Given that the alkaline-treated zein under applied US energy of 10 and 30 kJ shows a molecular mass distribution very similar to that of zein treated in NaOH without US, this latter approach with no applied US was chosen in the synthesis of zein-LDH biohybrids. In addition, the treatment combining alkaline medium and the applied US energy of 60 kJ was also selected for comparison, as it provided the smaller fractions of zein. As explained below, the FTIR and ^13^C NMR spectra of the alkaline-treated zein prepared by applying 60 kJ US (see below), indicate that amide I band is preserved, which suggest that the secondary structure of protein (α-helix) remains unaltered after the US treatment, corroborating the SDS-PAGE results (Figure 3).

### 3.1. Zein-Layered Double Hydroxides Biohybrids

**Figure 4:**
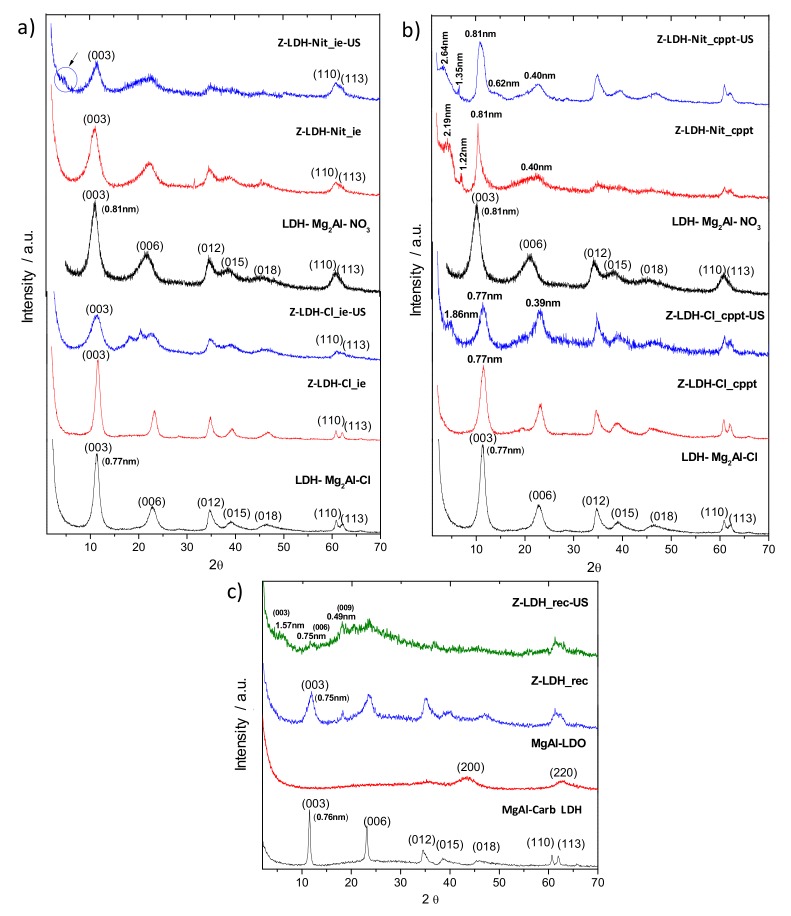
XRD diffractograms of Z-LDH biohybrids prepared by the (**a**) ion-exchange, (**b**) co-precipitation, and (**c**) reconstruction methods from zein dissolved in 0.1 M NaOH with and without US (60 kJ).

The LDH involving Mg and Al metals in a 2:1 MgAl ratio and containing chloride, nitrate or carbonate as interlayer anions were chosen for this study, as they are probably the most studied LDH materials. Three different methods of synthesis (ion-exchange, co-precipitation and reconstruction) were explored with the aim to prepare the corresponding zein-layered double hydroxide biohybrids (Z-LDH) from zein solubilized in 0.1 M NaOH, under magnetic stirring or US. Mg–Al LDH were synthesized with Cl^−^ (MgAl–Cl) or NO_3_^−^ interlayer anion (MgAl–Nit) showing as theoretical formula [Mg_0.67_Al_0.33_(OH)_2_]Cl_0.33_·nH_2_O and [Mg_0.67_Al_0.33_(OH)_2_]_2_(NO_3_)_0.33_·nH_2_O, respectively. The content of Mg and Al was analyzed in both samples by EDX in several areas of the solids, finding that the 2:1 Mg:Al ratio corresponds to that expected in both compounds. Figure 4 shows the diffractograms of the starting LDH samples. The basal distances calculated by the Bragg’s equation for MgAl–Cl and MgAl–Nit LDHs from the 2 theta angle of the (*003*) reflection, are 0.77 nm and 0.82 nm for MgAl–Cl and MgAl–Nit, respectively, and coincide with the values reported in the literature for the MgAl-LDH materials containing chloride and nitrate anions [47].

The X-ray diffractograms of the biohybrids prepared by ion-exchange of Cl^−^ or NO_3_^−^ by alkaline-treated zein under magnetic stirring or by application of US (Figure 4a) show the characteristic (*012*), (*018*), (*110*) and (*113*) reflections, indicative of the preservation of the LDH structure. XRD patterns very similar to that of the pristine LDH are observed in both biohybrids, suggesting that the ion-exchange reaction does not take place, regardless of the treatment used to dissolve zein or the type of anion present in the interlayer. However, a careful analysis of the diffractogram of the biohybrid prepared from MgAl–Nit and zein treated under US (60kJ), Z-LDH-Nit_ie-US, reveals a slight shoulder at 2θ of 4.4°, corresponding to a basal spacing of 2.0 nm, (arrow in Figure 4a). This shoulder would point to the presence of a phase with intercalated zein. Considering that the thickness of a brucite-type layer is approximately 0.48 nm [48], the increment of the interlayer distance of the (*003*) reflection for the Z-LDH based on MgAl–Cl and MgAl–Nit corresponds to 0.29 nm and 0.33 nm, respectively. These values are in agreement with the size of the intercalated Cl^−^ and NO_3_^−^ ions [49], suggesting that, in both cases, zein is not located in the interlayer space of the LDH structure.

Co-precipitation method was another strategy employed in this study in order to effectively obtain a zein intercalated phase into the LDH solid. In this case, the XRD patterns of the Z-LDH systems prepared by co-precipitation reaction show that there are no significant differences between the diffractogram of the Z-LDH-Cl biohybrid prepared with zein dissolved by magnetic stirring (Z-LDH-Cl_cppt) and that of the pristine MgAl–Cl (Figure 4b). This result suggests that the protein could be adsorbed on the external surface of the MgAl LDH structure. Conversely, a new peak at 4.8 degrees of 2θ can be identified in the diffractogram of the material prepared from zein dissolved under US, corresponding to a basal spacing of 1.85 nm (Z-LDH-Cl_cppt-US, Figure 4b). This new peak at lower 2θ values than the (*003*) reflection of MgAl LDH can be attributed to a phase of intercalated zein. In the case of materials prepared from the nitrate salts, the presence of two peaks at lower 2θ than the (*003*) reflection of LDH is observed in the XRD patterns of Z-LDH-Nit_cppt and Z-LDH-Nit_cppt-US (Figure 4b), which were prepared from zein solubilized in alkaline media using magnetic stirring or US, respectively. Considering the first peak in the corresponding XRD patterns of these biohybrids, the determined basal spacing values were 2.15 nm for Z-LDH-Nit_cppt, and about 2.67 nm for Z-LDH-Nit_cppt-US, respectively. Thus, the basal spacing increase values in the Z-LDH-Cl_cppt-US, Z-LDH-Nit_cppt and Z-LDH-Nit_cppt-US biohybrids can be calculated as 1.38 nm, 1.84 nm and 2.19 nm, respectively. Despite the presence of intercalated phases in three of the four Z-LDH biohybrid systems prepared by co-precipitation, the most intense peak still corresponds in all cases to the (*003*) reflection due to non-intercalated MgAl-LDH, revealing that these biohybrids comprise intercalated and non-intercalated phases, with a higher content in the last one. 

The third attempt in the preparation of Z-LDH biohybrids was the reconstruction method, and their respective XRD patterns are shown in Figure 4c. In this method, a MgAl LDH containing carbonate ions in the interlayer region (MgAl-Carb) was firstly synthesized and calcined at 350 °C to produce the corresponding Mg–Al layered double oxide (LDO), from which the LDH structure is reconstructed by hydration of the LDO using a solution containing negatively charged zein. The diffractogram of the pristine MgAl-Carb LDH shows the characteristic reflections of LDH, with the highest intensity peak corresponding to the (*003*) plane. From this reflection, a basal distance of 0.76 nm was determined, matching the reported values for this LDH containing carbonate anions [47,50]. It is not possible to detect any of the typical peaks of the LDH in the XRD pattern of the MgAl-LDO obtained from calcination of MgAl-Carb LDH, but only those attributed to amorphous MgO (periclase) [50,51]. Reconstruction of the LDH structure using a magnetically stirred zein solution (Z-LDH_rec) is confirmed by the presence of the (*110*) and (*113*) reflections, at the same typical angles observed in the starting MgAl LDH structure. In the diffractogram of the reconstructed material, the (*003*) reflection peak appears at slightly lower 2θ angles than in the pristine MgAl-Carb LDH, indicating that zein was not intercalated between the LDH layers. Considering that the reconstruction is carried out in CO_2_-free water, the LDH layer charge was probably compensated by OH^−^ anions, giving rise to a phase known as meixnerite [48]. In the case of the Z-LDH_rec-US biohybrid, the diffractogram is poorly defined, showing the presence of scatter between 20–30° in 2θ that could indicate the presence of amorphous zein. In this diffractogram, the (*110*) and (*113*) characteristic reflections of the LDH structure can be appreciated together with new peaks related to basal spacing values of 1.54 nm, 0.75 nm, and 0.50 nm, which can be assigned to (*003*), (*006*), and (*009*) reflections, respectively. From those values, it is possible to calculate an average basal spacing of 1.51 nm, which confirms the presence of zein intercalated in the reconstructed LDH. Considering that a brucite layer thickness is 0.48 nm, the increment in the interlayer spacing in the Z-LDH_rec-US biohybrid is 1.03 nm.

The zein content in all the biohybrids was calculated from CHNS chemical analysis (Table 1) and reveals that the amount of zein in the LDH is variable, not only depending on the anion (chloride or nitrate) used in the LDH preparation, but it also seems to depend on the methods used for zein solubilization and for preparation of the biohybrids. According to these results, the samples prepared by co-precipitation showed higher zein content compared to the other two approaches. The Z-LDH-Nit_cppt-US biohybrid showed the highest amount of zein (58 g of Z per 100 g of LDH). The fact that zein ultrasonication favors its disaggregation and increases the small protein fractions, as revealed by SDS-PAGE results (Figure 3), could enable the partial intercalation of zein in Z-LDH-Cl_cppt-US and Z-LDH-Nit_cppt-US, which indicates the treatment provokes the preferential adsorption of some fractions of zein with different C/N ratio. The fact that nitrate ions show lower stability than chloride as interlayer anions may also facilitate the intercalation of zein in the case of Z-LDH-Nit_cppt and Z-LDH-Nit_cppt-US samples. However, the co-precipitation method seems to be only partially efficient to achieve biohybrids where the protein is completely intercalated into the LDH. A similar effect of the disaggregation of the protein under US treatment seems to facilitate the intercalation of zein in the biohybrids prepared by reconstruction, revealing for this case a zein content of 36 g per 100 g of LDH. In contrast, the Z-LDH-Nit_ie-US prepared by ion-exchange reaction showed only 13.0 g of zein/100 g of MgAl-LDH, indicating that intercalation of zein in LDH by this process is very difficult, most likely requiring long reaction times or to carry out the process into several steps. Given that zein was not successfully intercalated by this strategy (ion-exchange), this method was discarded for further studies. However, it is important to highlight that it is difficult to ascertain if the content of the zein present in the intercalation compounds is adsorbed at the external surface of the inorganic solid, especially because it just produced a partial intercalation.

**Figure 5:**
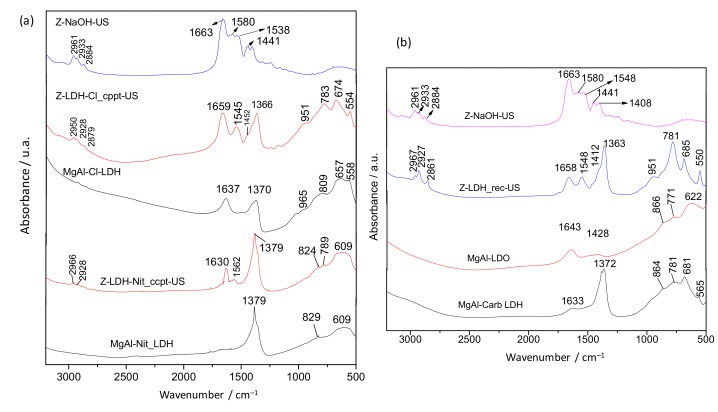
FTIR spectra in the 3200–500 cm^−1^ region of LDH and Z-LDH biohybrids prepared by (**a**) co-precipitation and (**b**) reconstruction from zein dissolved in 0.1 M NaOH under US.

The interaction between sonicated zein and MgAl LDH in the biohybrids prepared by co-precipitation and reconstruction was investigated by FTIR spectroscopy (Figure 5). The FTIR spectra of the biohybrids prepared by co-precipitation (Figure 5a), show the typical absorption bands between 600–400 cm^−1^ ascribed to the deformation vibration modes of metal-oxygen bonds in the LDH layers [52,53]. The vibrational bands between 2915 and 2848 cm^−1^ are attributed to the ν_C–H_ vibration of CH_2_ groups from the protein. In the Z-LDH-Cl_cppt-US and Z-LDH-Nit_cppt-US biohybrids (both shown in Figure 5a), the band at 1659 and 1630 cm^−1^, respectively, which are assigned to the amide I, are shifted toward lower wavenumber compared to that observed in the Z-NaOH-US spectrum (1663 cm^−1^). Interestingly, the frequency of the amide II characteristic vibration band, which appears as a single band at around 1545 cm^−1^ in the Z-LDH biohybrids, is also shifted. The spectrum of the Z-LDH-Nit_cppt-US biohybrid shows also a band at 1379 cm^−1^, which is attributed to nitrate ions of the non-intercalated LDH-phase [54]. The band at 1370 cm^−1^ observed in the MgAl–Cl spectrum, is most likely due to the ν_3_ vibrational mode of carbonate anions, which could be incorporated during the LDH synthesis from atmospheric CO_2_ in spite of carrying out the synthesis under N_2_ flow [55]. Figure 5b shows the FTIR spectra of the Z-LDH_rec-US biohybrid, Z-NaOH-US, the starting MgAl-CO LDH and the MgAl-LDO material. These spectra reveal that the band at 1663 cm^−1^ in Z-NaOH-US, assigned to ν_CO_ vibrations of C=O of amide I, is slightly shifted toward a lower wavenumber in the Z-LDH_rec-US biohybrid, appearing at 1658 cm^−1^. All these shifts observed in the biohybrids prepared here, may be related to interactions established between the negatively charged glutamate groups of the alkaline-treated zein and the LDH host, as reported by Charradi and co-authors [5] in studies about the immobilization of hemoglobin in MgAl-LDH. In the spectrum of the Z-LDH_rec-US biohybrid, the band ascribed to the ν_asym C–O_ stretching mode of the carbonate anion at 1363 cm^−1^ can be also distinguished. The vibrational bands between 2915 and 2848 cm^−1^ are attributed to ν_C–H_ vibration of CH_2_ groups from zein, and the set of small bands below 1000 cm^−1^ region are ascribed to the host inorganic lattice vibrations (stretching M–O–M) of the LDH structure [31]. 

**Figure 6:**
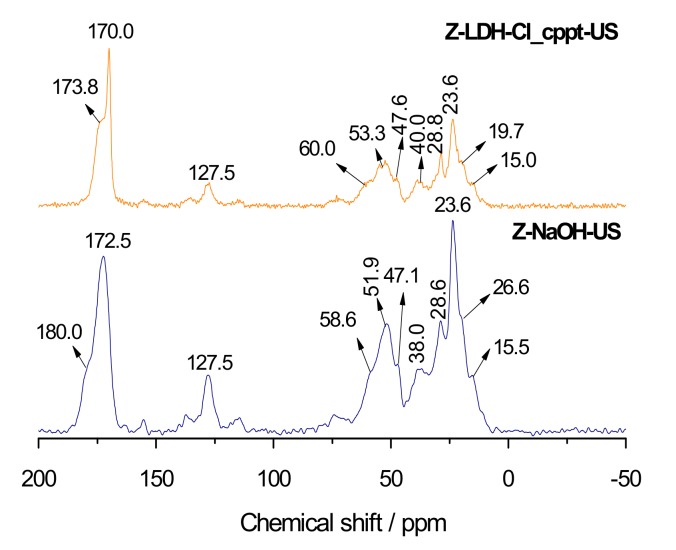
Solid state ^13^C NMR spectra of Z-NaOH-US and the Z-LDH-Cl_cppt-US biohybrid.

The interactions between zein and the layered solid pointed out by FTIR measurements (Figure 5) can be confirmed by solid state ^13^C NMR spectra of Z-NaOH-US and Z-LDH-Cl_cppt-US biohybrid (Figure 6). Both spectra are very similar and the main differences are related to the signal due to the carbonyl carbons. This signal appears at 172.5 ppm in the spectrum of the starting zein, showing a shoulder at 180 ppm, but both are shifted toward lower magnetic fields in the spectra of the biohybrids, appearing at 170 ppm and 173 ppm, respectively, in the case of Z-LDH-Cl_cppt-US. The small shift of both signals reflects a weaker interaction between the negatively charged groups present in biopolymer and the charged LDH layers acting as the positive counterions, which is in agreement with the results obtained from FTIR discussed above. Another evidence of the intercalation process of zein into LDH is the thermal behavior of these biohybrid materials in comparison to pure Z-NaOH-US (Figure S1). In this case, a clear improvement of the thermal stability of the protein moiety was evidenced, where the decomposition of zein increased up to temperatures around 400 °C in the hybrid materials. 

**Figure 7:**
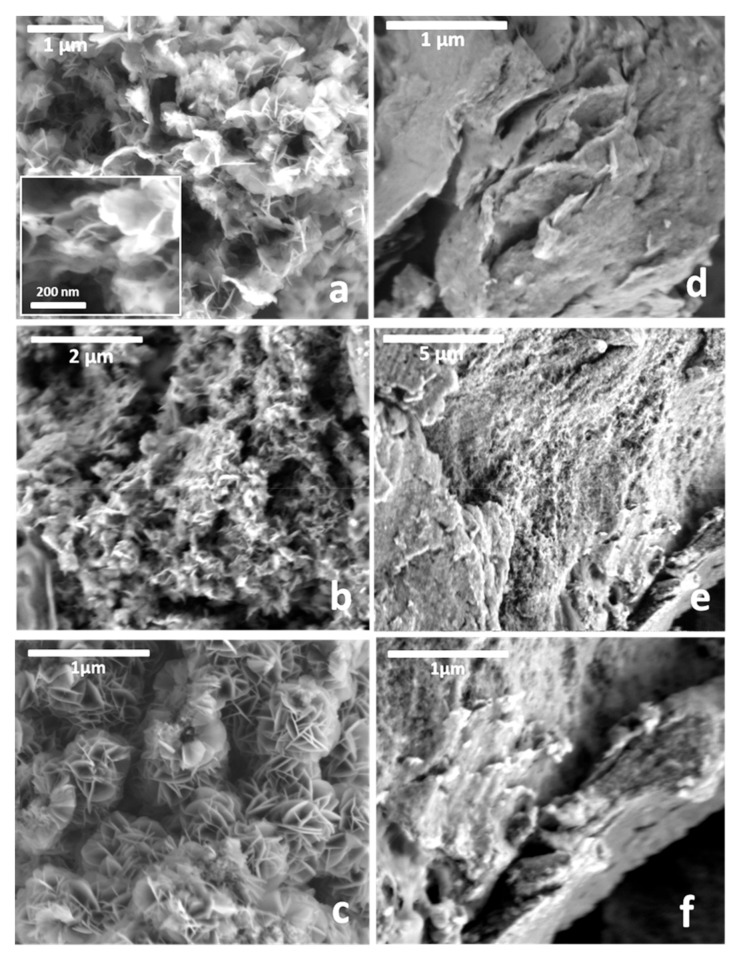
FE-SEM images of (**a**) MgAl–Cl, (**b**) MgAl–Nit and (**c**) MgAl-Carb LDH, as well as the (**d**) Z-LDH-Cl_cppt-US, (**e**) Z-LDH-Nit_cppt-US, and (**f**) Z-LDH_rec-US materials.

LDH hosts as well as their resulting biohybrids prepared by co-precipitation and reconstruction procedures were observed by FE-SEM (Figure 7). Images of the MgAl–Cl, MgAl–Nit, and MgAl-Carb LDH (Figure 7a–c, respectively) show the typical “sand-rose” morphology of many LDH materials [56]. The Z-LDH biohybrids show a quite different aspect (Figure 7d–f). The presence of zein in the synthesis medium seems to give rise to the aggregation of the lamellar LDH particles, providing compactness to the resulting materials, probably cemented by the presence of zein. Similar morphologies were observed in other hybrid systems prepared also from the combination of LDH with biomolecules, such as alginate and ι-carrageenan polysaccharides [25].

### 3.2. Zein-Layered Single Hydroxide Biohybrids

**Figure 8:**
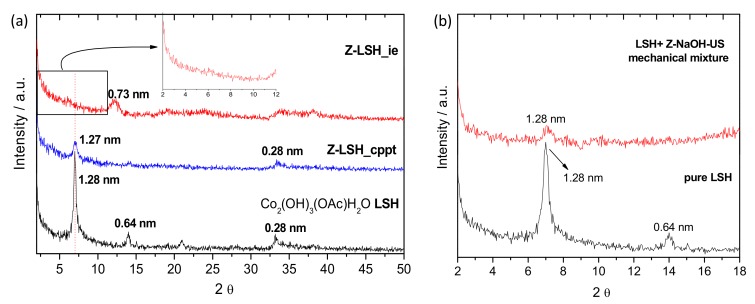
(**a**) XRD patterns of pristine Co-LSH, and Z-LSH_cppt and Z-LSH_ie biohybrids prepared by co-precipitation and ion-exchange methods, respectively; (**b**) XRD patterns of pristine Co-LSH containing acetate anions and the mechanical mixture based on Co-LSH and Z-NaOH-US.

The preparation of zein-based biohybrids by intercalation into layered single hydroxide (LSH) with acetate anions in the interlayer region (Co_2_(OH)_3_(CH_3_COO)·H_2_O) was also explored. Based on the previous results on zein intercalation in LDH (*vide supra*) showing that zein dissolved in NaOH solution under US (60 kJ) was more easily intercalated due to the presence of smaller protein fractions, the same solution was chosen in the preparation of LSH biohybrids employing the co-precipitation and ion-exchange procedures. Figure 8 displays the XRD patterns of the pristine Co-LSH and the biohybrids prepared by co-precipitation (Z-LSH_cppt) and ion-exchange (Z-LSH_ie) methods. From Figure 8a, it is possible to observe that in the diffractogram of the biohybrid prepared by co-precipitation (Z-LSH_cppt) the peak at 6.0° in 2θ, related to the (*003*) reflection, appears at the same position than in the diffractogram of the pristine LSH. In both materials, a basal spacing of 1.27 nm was determined. Considering that the thickness of the hydroxide layer constituted by the cobalt triple deck layers is ~0.6 nm [57], it could be deduced a basal increment of 0.67 nm, compatible with the acetate anions size [49]. On the other hand, the diffractogram of the Z-LSH_ie biohybrid does not show any reflection in the 2° to 10° range in 2θ (Figure 8a). Conversely, a new peak is observed at approximately 12.2° in 2θ, which corresponds to a basal spacing of 0.73 nm. According to Laget et al. [58] and Si et al. [36], when the OH/exchanged anion ratio deviates from 3, the exchange reaction is not topotactic, and a dissolution–recrystallization processes may take place. Thus, a possible delamination of the structure could be also taking place in the Z-LSH_ie material. In order to support this finding, a mechanical mixture of the dried Z-NaOH-US and LSH was prepared using the same amounts than in the ion-exchange reaction. The diffractogram of this mechanical mixture (Figure 8b) still shows the peak of the starting LSH. This result discards a possible dilution effect in the XRD pattern of Z-LSH_ie (Figure 8a) and confirms that the observed changes could be attributed to the exfoliation of the LSH sheets due to the incorporation of the protein, as indicated above. This sample has a zein content of 11.4 g of zein/100 g of Co-LSH, determined by elemental chemical analysis. The cobalt-based layered hydroxide seems to be more susceptible to exfoliation according to various studies, as for instance those about hybrids prepared by ion-exchange reactions of Co–Ni LDH with formamide [59] or by assembling Co–Al LDH and carbon nanotubes through electrostatic forces [60]. 

**Figure 9:**
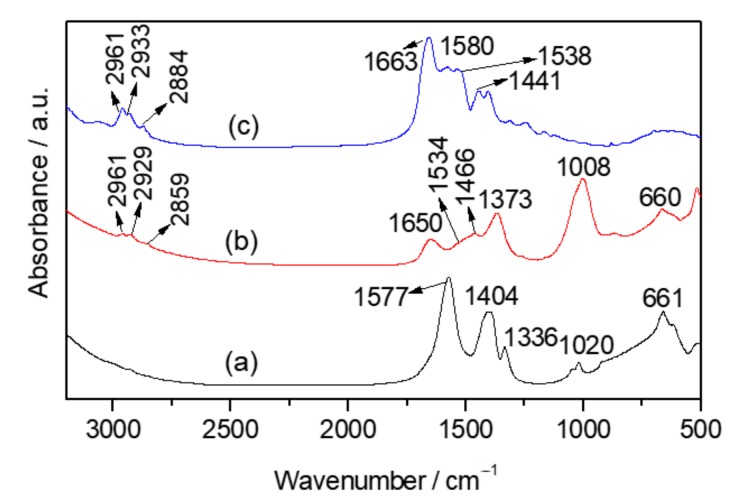
FTIR spectra in the 3200–500 cm^−1^ region of pristine (**a**) Co_2_(OH)_3_(CH_3_COO)-LSH, the (**b**) Z-LSH_ie biohybrid. and the (**c**) Z-NaOH-US.

FTIR spectroscopy was used to examine the possible interactions between the protein and the Co-LSH host in the Z-LSH_ie biohybrid (Figure 9). Together with the bands below 800 cm^−1^ assigned to the metal-oxygen vibrations of the Co-LSH framework, also observed in the spectrum of the pristine LSH (Figure 9 a), the spectrum of Z-LSH_ie shows other specific bands between 2970–2850 cm^−1^ associated with ν_C-H_ vibration mode of the biopolymer (Figure 9b). In this last spectrum, the shifts in the bands at 1650 cm^−1^ and 1534 cm^−1^ ascribed to the C=O and C–N vibration modes can be detected in comparison to those of the Z-NaOH-US spectrum (Figure 9c). These differences could indicate the existence of interactions between the biomacromolecule and the LSH, as explained in the characterization of Z-LDH biohybrids (Figure 5). In the case of LSH, the presence of the functional groups of the intercalated anion (e.g., carboxylate groups from the acetate species) may help to favor the interaction of the protein producing different hybrid structures (e.g., exfoliated). In fact, the antisymmetric and symmetric stretching vibration modes of COO^−^ groups, appearing at 1577 cm^−1^ and 1404 cm^−1^, respectively in the pristine LSH (Figure 9a), disappear in the spectrum of the biohybrid material, indicating the removal of the acetate anion from the LSH. 

**Figure 10:**
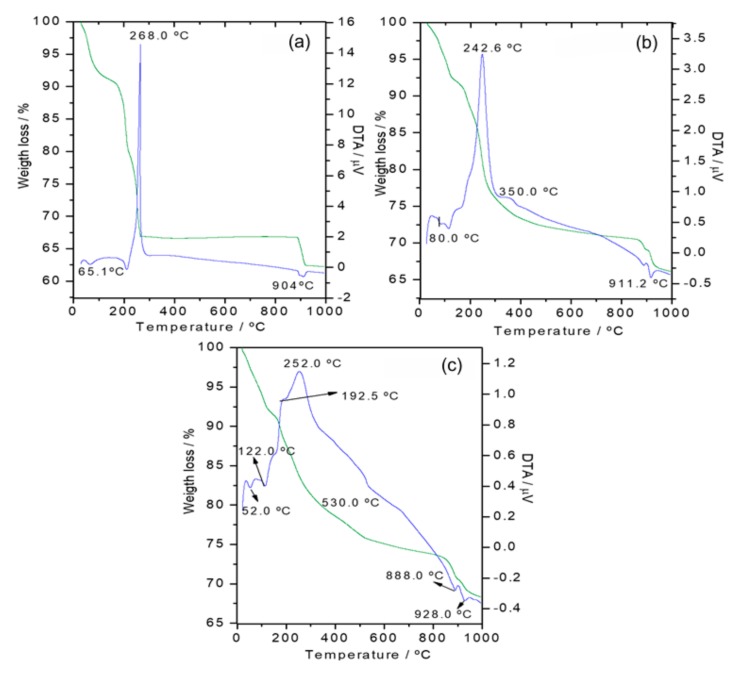
TG and DTA curves of (**a**) Co-LSH, and (**b**) Z-LSH_cppt and (**c**) Z-LSH_ie biohybrids carried out under air flow.

TG and DTA curves of the Co-LSH and the Z-LSH biohybrids obtained under air flow are shown in Figure 10. All the curves show weight losses in the 25–200 °C and 900–1000 °C ranges, which are related to the removal of physically adsorbed water molecules and to the condensation of the structural hydroxyl groups of the LSH layers, respectively. Pristine Co-LSH and Z-LSH_cppt biohybrid show a similar thermal behavior, with a strong exothermic event at the 200–370°C temperature range, which is attributed to the removal of the interlayer acetate anions, confirming that zein was not intercalated in this biohybrid. The Z-LSH_ie biohybrid presents TG and DTA profiles very different to those of the LSH and the Z-LSH_cppt biohybrid. Various mass losses are observed above 200 °C, which are accompanied by exothermic events in the DTA curves. The weight loss in the 200–400 °C range can be attributed to the partial decomposition of the biopolymer and the removal of remaining acetate anions. The total weight loss associated with processes at temperatures between 350 °C and 900 °C may be due to the final decomposition of the zein associated with the layered solid in different arrangements, as observed in studies of biohybrids based on zein and clays [61].

**Figure 11:**
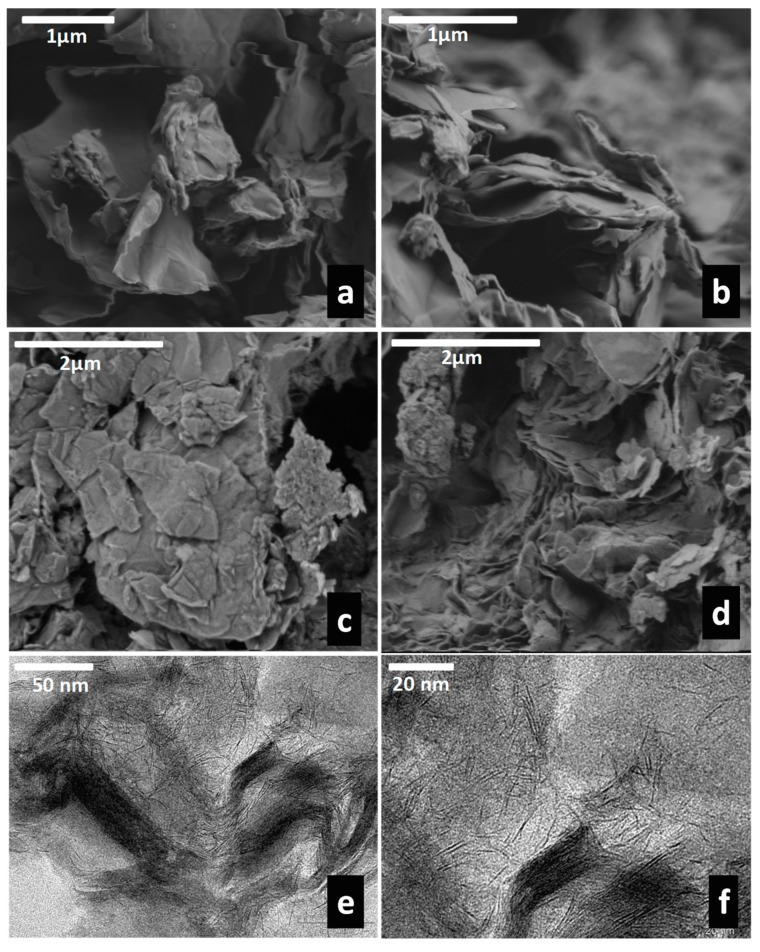
FE-SEM images of the pristine Co-LSH (**a**,**b**), and the Z-LSH_ cppt (**c**) and Z-LSH_ie (**d**) biohybrids. TEM images of Z-LSH_ie biohybrid (**e**,**f**).

FE-SEM images the pristine Co-LSH material (Figure 11a,b) and the Z-LSH_cppt biohybrid (Figure 11c) show similar morphologies, consisting of thin platelet–shaped microcrystals due to the LSH material. In constrast, the Z-LSH_ie biohybrid shows a more compact morphology (Figure 11d), similar to related peptides-Co-LSH materials reported by Si and co-authors [36]. The Z-LSH_ie sample was analyzed with more detail by TEM, and the obtained images (Figure 11e,f) clearly show the presence of highly exfoliated Co-LSH together with some intercalated phases, corroborating the XRD results.

## 4. Conclusions

An initial investigation on the preparation of biohybrids based on the corn protein zein and the inorganic host materials MgAl layered double hydroxide and Co_2_(OH)_3_ layered single hydroxide was carried out following various synthetic approaches. The results shown here demonstrate the possibility to intercalate zein, at least partially in certain cases, in both LDH and LSH host solids. The nature of the interlayer anions in LDH can have strong influence on the assembly to zein, resulting in different biohybrid structures. The solubilization of zein in alkaline medium (0.1 M NaOH) and applying US irradiation seems to favor its incorporation between the LDH layers, which is attributed to the fragmentation of the protein agglomerates and to the presence of protein fractions of reduced molecular mass. Using sonicated zein, the biohybrids prepared by co-precipitation method are composed of mixed phases with partial intercalation, while the reconstruction method allowed the preparation of a single phase with completely intercalated zein but showing low crystallinity. In the zein–LDH hybrids, the partial accommodation of zein implies electrostatic interactions with the inorganic host as suggested by FTIR and NMR studies. In the case of biohybrids based on Co-LSH prepared by the ion-exchange method, a highly exfoliated intercalated phase was obtained. In this case, the presence of the carboxylate groups from the acetate species located in the interlayer region of the LSH could help to favor the interaction of the protein promoting an exfoliated structure. Thus, this preliminary study has shown the possibility to prepare zein-layered hydroxides biohybrids of interest for applications in biomedicine, biosensing or as components for electronic devices. However, a further deep study of the properties should be carried out in view to clearly identify the possible applications of these biohybrids. 

## Figures and Tables

**Table 1:** Zein content in Z-LDH biohybrids prepared by different methods. The amount of zein was calculated from the content of carbon in the samples.

Z-LDH Biohybrid Samples	Carbon Content	Nitrogen Content	C/N Ratio	Zein Content (g Z/100 g of LDH)
Z-LDH-Nit_ie-US	5.16	2.02	2.55	13.0
Z-LDH-Cl_cppt-US	15.2	4.71	3.22	40.0
Z-LDH-Nit_cppt	16.1	5.12	3.14	43.2
Z-LDH-Nit_cppt-US	21.3	5.54	3.84	57.5
Z-LDH_rec-US	13.6	3.82	3.56	36.0

## Supplementary Materials

The following are available online at https://www.mdpi.com/1996-1944/13/4/825/s1, Figure S1. TG and DTA curves of Z-NaOH-US, and the Z-LDH-Cl_cppt-US and Z-LDH-Nit_cppt-US bio-hybrids under air flow.
