# Supplementary material for: Zein-Layered Hydroxide Biohybrids: Strategies of Synthesis and Characterization
Source: Materials (Basel). 2020 Feb 11;13(4):825. doi: 10.3390/ma13040825 (PMC7079599; doi:10.3390/ma13040825)
Supplement: Supplementary file 1 [file materials-13-00825-s001.pdf]

Supplementary information

Zein-layered hydroxide biohybrids: strategies of synthesis and characterization by Ana C. S. Alcântara, Margarita Darder, Pilar Aranda, and Eduardo Ruiz-Hitzky.

The Figure S1, shows the TG and DTA curves of Z-NaOH-US and the Z-LDH-Cl_cppt-US and Z-LDH-Nit_cppt-US (*) bio-hybrids in the range of 25-100ºC temperature, obtained under air flow. Several steps of decomposition are observed in the case of Z-NaOH-US, which could correspond to pyrolysis events at temperatures below 430ºC and to the decomposition and combustion of the protein at temperatures higher than 500ºC. The thermal behavior of the Z-LDH-Cl_cppt-US and Z-LDH-Nit_cppt-US bio-hybrids is very different (Figure S1). In both cases, the thermal stability of zein is increased up to temperatures around 400 °C. From the TG/DTA curves of Z-LDH-Cl_cppt-US and Z-LDH-Nit_cppt-US bio-hybrids, weight losses up to 200 ºC are determined, being 10 and 12% respectively, , which are related to the elimination of water molecules. Above 200 °C both bio-hybrids undergo thermal decomposition in two main stages: i) between 200 ºC and 450 ºC, where a partial elimination of zein occurs, being associated which the exothermic peaks at 388 and 355 ºC, for Z-LDH-Cl_cppt-US and Z-LDH-Nit_cppt-US, respectively; and ii) between 450 ºC and 600 ºC, where the combustion of the organic matter associated with the exothermic processes is completed. In this temperature range, the dehydroxylation of the LDH and the elimination of residual chloride or nitrate anions also occur.

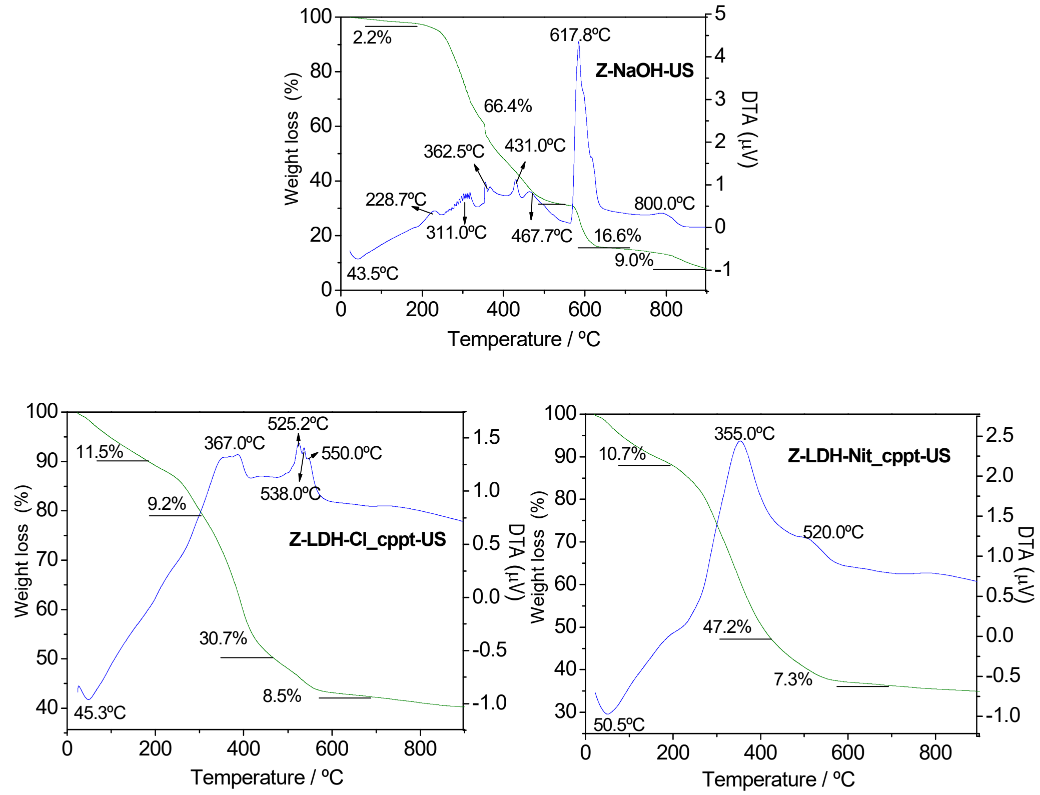

Figure S1. TG and DTA curves obtained in air flow of Z-NaOH-US, and the Z-LDH-Cl_cppt-US and Z-LDH-NO_cppt-US bio-hybrids.

(*) Note: the nomenclature of samples is detailed in the main part of the article. For instance, the biohybrid materials synthesized by the co-precipitation method derived from MgAl-Cl and MgAl-Nit LDH and using zein dissolved by magnetic stirring were denoted as Z-LDH-Cl_cppt and Z-LDH-Nit_cppt, respectively, while those obtained from sonicated zein were labeled as Z-LDH-Cl_cppt-US and Z-LDH-Nit_cppt-US, respectively.
